# Ability of Large Language Models to Answer Patients’ Questions and Generate Educational Materials for Uncommon Retinal Conditions

**DOI:** 10.1177/24741264261469005

**Published:** 2026-08-18

**Authors:** Samuel A. Cohen, Prashant D. Tailor, Adrian Au, Jennifer Vu, Alejandro I. Marin, Pradeep S. Prasad, Julie Kwon, Hamid Hosseini, Jayanth Sridhar

**Affiliations:** 1 Department of Ophthalmology, UCLA Jules Stein Eye Institute, University of California Los Angeles, Los Angeles, CA, USA; 2 Division of Ophthalmology, Olive View-UCLA Medical Center, University of California Los Angeles, Sylmar, CA, USA

**Keywords:** retina, large language model, artificial intelligence, accuracy, readability, ChatGPT, Google, Copilot

## Abstract

**Purpose:**

To assess the ability of large language models to accurately and comprehensively respond to frequently asked questions and generate patient education materials related to uncommon retinal conditions.

**Methods:**

A total of 50 frequently asked questions related to 10 uncommon retinal conditions were input into 3 large language models: ChatGPT-4o1, Google Gemini 2.0 Flash, and Microsoft Copilot (updated January 7, 2025). The accuracy and completeness of responses to frequently asked questions were evaluated by retina specialists using a Likert scale ranging from 1 (very inaccurate/not at all complete) to 5 (very accurate/completely complete), while readability was assessed using validated indices. The large language models were also instructed to generate patient education materials at specific reading levels, which were again evaluated for accuracy, completeness, and readability.

**Results:**

Responses to patient frequently asked questions were written at mean grade levels of 15.3 ± 1.4 (ChatGPT), 15.7 ± 1.9 (Gemini), and 14.6 ± 1.3 (Copilot), respectively (*P =* .02). Mean accuracy scores were 4.02 ± 0.7, 4.16 ± 0.7, and 3.81 ± 0.8. Accuracy scores for large language models-generated patient education materials were 4.70 ± 0.7 (ChatGPT), 4.85 ± 0.4 (Gemini), and 4.30 ± 0.8 (Copilot). When instructed to revise educational materials to improve understandability, all large language models significantly reduced reading levels by 4.8 (ChatGPT), 6.9 (Gemini), and 3.8 (Copilot) grade levels (*P* < .001) without compromising accuracy.

**Conclusions:**

Large language models can accurately respond to patients’ frequently asked questions related to uncommon retinal conditions. Furthermore, large language models can effectively improve the readability of existing education materials for patients with varying levels of health literacy. A deeper understanding of large language model applications may facilitate their integration into clinical practice.

## Introduction

In recent years, large language models have surged in popularity as a source of health information, with more than half of Americans now using large language models in their daily lives.^1–3^ While ChatGPT and Google Gemini are among the most widely used large language models, Microsoft Copilot has also been shown to effectively counsel patients.^4–7^

Despite their increasing popularity, evidence regarding the ability of large language models to accurately respond to patients’ frequently asked questions and provide patient counseling remains mixed. Within ophthalmology, some studies have supported the use of large language models for patient education, while others have claimed that large language models provide inaccurate, potentially harmful information.^8–13^ Furthermore, prior studies examining the use of large language models for patient education in retina have focused primarily on common retinal conditions.^14–16^ Consequently, there is a paucity of data regarding the ability of large language models to respond to patients’ frequently asked questions and generate patient education materials for uncommon retinal conditions, for which high-quality, comprehensible online resources are often limited.^17–19^ Furthermore, large language models are frequently updated, and there have been few studies evaluating the patient education capabilities of the most current large language model versions examined in the present study.

As such, this study had 2 primary objectives. First, a panel of fellowship-trained vitreoretinal surgeons evaluated and compared the accuracy and readability of responses to patients’ frequently asked questions related to uncommon retinal conditions generated by 3 large language models. Second, we assessed the ability of these models to generate accurate and comprehensive patient education materials at various reading levels upon request, with the goal of providing customized educational resources for patients with varying health literacy levels.

## Methods

All study protocols adhered to the Declaration of Helsinki. Verbal informed consent to participate in this study was provided by all expert panelists, described as follows.

### Selection of and Responses to Frequently Asked Questions

For all Google searches used in data collection, a clean installation of Google Chrome was used in Incognito mode. Sponsored results and location filters were disabled to eliminate bias from prior search activity and geographically targeted search results. Ten search terms were selected based on recent review articles discussing uncommon retinal conditions and expert consensus regarding uncommon retinal diseases encountered in clinical practice.^20,21^ The selected conditions were retinitis pigmentosa, choroideremia, Stargardt disease, cone-rod dystrophy, Leber congenital amaurosis, achromatopsia, retinoschisis, retinoblastoma, retinopathy of prematurity, and Best disease.

For all 10 search terms, a Google search was executed on January 5, 2025, and the first 5 frequently asked questions associated with each respective search term were noted. These 50 frequently asked questions were subsequently entered into 3 large language models—ChatGPT-4o1, Google Gemini 2.0 Flash, and Microsoft Copilot (last updated January 7, 2025)—on January 16, 2025, using a zero-shot prompting approach without prior training. A new conversation window was used for each question. Responses were recorded and subsequently analyzed for readability, accuracy, and completeness.

### Readability Analysis of Patients’ Frequently Asked Questions

The readability of responses to frequently asked questions generated by the large language models was assessed using 5 validated readability metrics: the Flesch Reading Ease score, Gunning Fog Index, Flesch–Kincaid Grade Level, Simple Measure of Gobbledygook, and Coleman–Liau Index. While the Flesch Reading Ease scale evaluates readability on a scale from 0 (very difficult) to 100 (very easy), the remaining indices estimate the grade level required to comprehend a given text. For example, a score of 7 indicates that the text is written at a seventh-grade (middle school) reading level. The Gunning Fog Index, Flesch–Kincaid Grade Level, Simple Measure of Gobbledygook, and Coleman Liau Index were averaged to generate a composite grade-level score for each response.

### Expert Panel Evaluation of Responses to Frequently Asked Questions

A panel of 3 fellowship-trained vitreoretinal surgeons independently reviewed responses to each of the 50 frequently asked questions generated by the 3 large language models in a masked, randomized manner. First, panelists evaluated the accuracy of each response using a Likert scale ranging from 1 (very inaccurate: substantial inaccuracies and high risk of harm) to 5 (very accurate: very good accuracy with no risk of harm). They then assessed the completeness of each response on a Likert scale from 1 (not at all complete) to 5 (completely complete). For responses deemed inaccurate or incomplete, panelists were asked to identify specific inaccuracies and missing information, respectively. The methodology used to evaluate the accuracy and completeness of large language model-generated responses was consistent with that used in several prior studies.^14,22–24^

### Generation of Patient Education Materials by Large Language Models

All 3 large language models were instructed to generate patient education materials for each of the 10 uncommon retinal conditions. The following prompt was entered into each large language model: “[Insert name of large language model], please produce education materials for patients and their families regarding [insert retinal condition]. Please provide an explanation of the disease, including its cause, symptoms, treatments, and prognosis. Please limit your response to 2 paragraphs.” Responses were recorded for analysis. Prompts were developed based on the format commonly used by existing patient education materials, such as those published by the American Academy of Ophthalmology.^25^ Responses were evaluated for readability using the 5 readability indices described earlier.

To assess the ability of large language models to generate educational materials at a specific reading level, each model was subsequently instructed to produce condition-specific patient education materials at a seventh-grade reading level, consistent with several previous studies.^8,26,27^ For these prompts, the following statement was added: “Please write these patient education materials at a seventh-grade reading level according to validated readability indices to ensure they are understandable to patients with lower health literacy.” The resulting responses were recorded and evaluated for readability.

### Expert Panel Evaluation of Patient Education Materials

A separate panel of 3 fellowship-trained vitreoretinal surgeons independently reviewed the patient education materials generated by the large language models for both accuracy and completeness using the same evaluation criteria described above to evaluate responses to frequently asked questions. In addition, for each of the 10 retinal conditions, the patient education materials generated at the seventh-grade reading level by each of the 3 large language models were listed side by side in a random order, and panelists were asked which version they would be most likely to use when providing educational materials to their patients.

### Statistical Analysis

The readability of large language model responses to patient frequently asked questions and large language model-generated patient education materials was compared using analysis of variance with post hoc Tukey honestly significant difference and 2-sample *t* tests, as appropriate. The accuracy and completeness of large language model responses to patients’ frequently asked questions and large language model-generated patient education materials were compared using Kruskal–Wallis tests with post hoc Dunn tests. The intraclass correlation coefficient was used to assess interrater agreement among expert panelists. As 12 statistical tests were conducted, a Bonferroni correction was applied, resulting in a threshold for statistical significance of *P* < .004 (.05/12).

## Results

Table 1 presents the 50 frequently asked questions identified through Google searches for the 10 retinal conditions included in this study.

**Table 1.** Most Frequently Asked Questions Regarding 10 Uncommon Retinal Conditions.

Uncommon Retinal Condition\Frequently Asked Questions	Retinitis Pigmentosa	Choroideremia	Stargardt Disease	Cone-Rod Dystrophy	Leber Congenital Amaurosis	Achromatopsia	Retinoschisis	Retinoblastoma	Retinopathy of Prematurity	Best Disease
Question 1	What is retinitis pigmentosa?	What is choroideremia?	What is Stargardt disease?	What is cone-rod dystrophy?	What is Leber congenital amaurosis?	What is achromatopsia?	What is retinoschisis?	What is retinoblastoma?	What is retinopathy of prematurity?	What is Best disease?
Question 2	What is the major cause of retinitis pigmentosa?	What are the symptoms of choroideremia?	Is Stargardt disease curable?	Can cone-rod dystrophy be cured?	Can Leber congenital amaurosis be cured?	Can achromatopsia be corrected?	How do you treat retinoschisis?	Can retinoblastoma be cured?	What is the main cause of retinopathy of prematurity?	Is Best disease curable?
Question 3	Can retinitis pigmentosa be reversed?	Is choroideremia curable?	What does a person with Stargardt disease see?	What is the prognosis for cone-rod dystrophy?	How is Leber congenital amaurosis treated?	What is the difference between cerebral achromatopsia and color blindness?	What is the difference between retinal detachment and retinoschisis?	What is the main cause of retinoblastoma?	What are the long-term effects of retinopathy of prematurity?	What are the symptoms of Best disease?
Question 4	What is the prognosis for retinitis pigmentosa?	What is the age of onset for choroideremia?	Can glasses help with Stargardt disease?	What is the difference between retinitis pigmentosa and code-rod dystrophy?	What are the symptoms of Leber congenital amaurosis?	Do people with achromatopsia see in black and white?	What should you avoid if you have retinoschisis?	Is chemotherapy effective for retinoblastoma?	How is retinopathy of prematurity treated?	Does Best disease cause blindness?
Question 5	At what age does vision loss occur in retinitis pigmentosa?	What is the prognosis for choroideremia?	What are the treatments for Stargardt disease?	What does a person with cone-rod dystrophy see?	When does Leber congenital amaurosis cause blindness?	What is the treatment for achromatopsia?	What is the prognosis for retinoschisis?	Do you lose your eye when you are diagnosed with retinoblastoma?	What are the 5 stages of retinopathy of prematurity?	What is the long-term prognosis for patients with Best disease?

Responses to patients’ frequently asked questions generated by ChatGPT, Google Gemini, and Microsoft Copilot were written at mean grade levels of 15.3 ± 1.4, 15.7 ± 1.9, and 14.6 ± 1.3, respectively (*F* = 6.67, *P =* .002). Responses generated by Google Gemini were written at a significantly higher grade level than those generated by Microsoft Copilot (*P* = .001). Mean Flesch Reading Ease scores for responses to frequently asked questions corresponded to a readability level ranging from “difficult to read” to “very difficult to read” across all 3 large language models (Table 2A).

**Table 2.** Readability of Responses to Patients’ Frequently Asked Questions and Large Language Model-Generated Patient Education Materials.

A. Readability of Responses to Patients’ Frequently Asked Questions						
Model	Flesch–Kincaid Reading Ease	Flesch–Kincaid Grade Level	Gunning Fog Index	Simple Measure of Gobbledygook	Coleman Liau Index	Average Grade Level
ChatGPT	29.4	14.2	17.9	12.8	16.4	15.3
Google Gemini	25.9	14.7	18.1	13.0	17.1	15.7
Microsoft Copilot	31.6	13.3	16.5	11.9	16.7	14.6
B. Readability of Large Language Model-Generated Patient Education Materials (Unspecified Reading Level)						
ChatGPT	31.1	13.7	17.7	12.7	16.4	15.1
Google Gemini	31.5	13.7	17.4	12.5	15.7	14.8
Microsoft Copilot	33.1	13.3	16.8	12.1	16.1	14.6
C. Readability of Large Language Model-Generated Patient Education Materials (Seventh-Grade Reading Level Requested)						
ChatGPT	60.6	9.1	11.6	8.4	11.9	10.3
Google Gemini	73.8	6.3	8.8	6.5	10.1	7.9
Microsoft Copilot	56.1	9.7	12.1	8.8	12.8	10.8

Expert panelists demonstrated strong agreement when evaluating large language model responses to frequently asked questions, with an intraclass correlation coefficient of 0.76. Mean accuracy scores (Likert scale, 1–5) for responses generated by ChatGPT, Google Gemini, and Microsoft Copilot were 4.02 ± 0.7, 4.16 ± 0.7, and 3.81 ± 0.8, respectively, corresponding to an accuracy level between “accurate” and “very accurate” for responses by ChatGPT and Google Gemini and between “moderately accurate” and “accurate” for Microsoft Copilot responses. Post hoc Dunn testing showed that responses generated by both ChatGPT and Google Gemini were significantly more accurate than those generated by Microsoft Copilot. Table 3 presents the mean accuracy scores of responses provided by large language models to patients’ frequently asked questions stratified by uncommon retinal condition.

**Table 3.** Mean Accuracy Level of Large Language Model Responses to Patients’ Frequently Asked Questions by Retinal Condition.

Model	Retinitis Pigmentosa	Choroideremia	Stargardt Disease	Cone-Rod Dystrophy	Leber Congenital Amaurosis	Achromatopsia	Retinoschisis	Retinoblastoma	Retinopathy of Prematurity	Best Disease
ChatGPT	4.40	3.80	4.40	4.13	4.00	4.13	3.13	4.00	4.00	4.27
Google Gemini	4.27	4.13	4.47	4.20	4.13	4.47	3.87	4.00	3.93	4.20
Microsoft Copilot	4.00	4.13	3.67	4.00	3.40	3.73	3.60	3.93	3.67	3.93
Mean	4.22	4.02	4.18	4.11	3.84	4.11	3.53	3.98	3.87	4.13

Mean completeness scores (Likert scale, 1–5) for responses to frequently asked questions generated by ChatGPT, Google Gemini, and Microsoft Copilot were 4.07 ± 0.7, 4.25 ± 0.7, and 3.62 ± 0.8, respectively, corresponding to a completeness level between “mostly complete” and “completely complete” for ChatGPT and Google Gemini responses and a completeness level between “somewhat complete” and “mostly complete” for Microsoft Copilot responses. Post-hoc Dunn testing showed that responses generated by ChatGPT and Google Gemini were significantly more complete than Microsoft Copilot responses. Table 4 displays the mean completeness scores of responses provided by large language models to patients’ frequently asked questions stratified by uncommon retinal condition.

**Table 4.** Mean Completeness Level of Large Language Model Responses to Patients’ Frequently Asked Questions by Retinal Condition.

Model	Retinitis Pigmentosa	Choroideremia	Stargardt Disease	Cone-Rod Dystrophy	Leber Congenital Amaurosis	Achromatopsia	Retinoschisis	Retinoblastoma	Retinopathy of Prematurity	Best Disease
ChatGPT	4.33	4.00	4.40	4.00	4.07	4.20	3.13	4.27	4.00	4.27
Google Gemini	4.27	4.40	4.47	4.27	4.27	4.47	3.80	4.27	4.07	4.27
Microsoft Copilot	3.67	3.80	3.73	3.73	3.13	3.73	3.20	3.53	3.80	3.87
Mean	4.09	4.07	4.20	4.00	3.82	4.13	3.38	4.02	3.96	4.14

Table 2B displays the readability metrics of large language model-generated patient education materials when no specific reading level was requested. ChatGPT, Google Gemini, and Microsoft Copilot generated education materials at mean grade levels of 15.1 ± 0.8, 14.8 ± 1.2, and 14.6 ± 1.3, respectively (*F* = 0.58, *P =* .564). The mean Flesch Reading Ease scores for the patient education materials corresponded to a readability level between “difficult to read” and “very difficult to read” for all 3 large language models (Table 2B).

Table 2C displays the readability metrics of large language model-generated patient education materials when a seventh-grade reading level was requested. ChatGPT, Google Gemini, and Microsoft Copilot generated educational materials at mean grade levels of 10.3 ± 1.0, 7.9 ± 0.8, and 10.8 ± 1.6, respectively (*F* = 16.81, *P* < .001). Post hoc Tukey honestly significant difference testing showed that educational materials generated by Google Gemini were written at a significantly lower reading level than those generated by ChatGPT and Microsoft Copilot. The mean Flesch Reading Ease scores ranged from “fairly difficult to read” for Microsoft Copilot-generated materials to “fairly easy to read” for Google Gemini-generated materials (Table 2C). Consistent with the grade-level analysis, post hoc Tukey honestly significant difference testing demonstrated that Google Gemini generated materials at a significantly lower reading level compared with ChatGPT and Microsoft Copilot. When instructed to revise patient education materials to improve understandability, all 3 large language models reduced reading levels by 4.8 grade levels (ChatGPT), 6.9 grade levels (Gemini), and 3.8 grade levels (Microsoft Copilot) (*P* < .001) without compromising accuracy.

Expert panelists showed strong agreement when evaluating large language model-generated patient education materials, with an intraclass correlation coefficient of 0.79. Mean accuracy scores (Likert scale, 1–5) for patient education materials generated at an unspecified reading level by ChatGPT, Google Gemini, and Microsoft Copilot were 4.70 ± 0.7, 4.85 ± 0.4, and 4.30 ± 0.8, respectively (Table 5). Mean completeness scores for these materials were 4.15 ± 0.6, 4.15 ± 0.5, and 3.95 ± 0.7, respectively (Table 5). When asked to identify inaccuracies and notable omissions in the large language model-generated patient education materials, panelists most frequently cited lack of content regarding gene therapy; insufficient explanations of technical terms such as “anti-VEGF”, “RPE”, and “cryotherapy”; and missing information regarding prognosis and long-term monitoring for several of the conditions studied.

**Table 5.** Accuracy and Completeness Scores of Large Language Model-Generated Patient Education Materials at Various Specified Reading Levels.

Model	Accuracy (Unspecified Reading Level)	Accuracy (Seventh-Grade Reading Level Requested)	Completeness (Unspecified Reading Level)	Completeness (Seventh-Grade Reading Level Requested)
ChatGPT	4.70	4.60	4.15	4.10
Google Gemini	4.85	4.55	4.15	4.00
Microsoft Copilot	4.30	4.10	3.95	3.80

Accuracy was rated on a 5-point Likert scale ranging from 1 (very inaccurate) to 5 (very accurate). Completeness was rated on a 5-point Likert scale ranging from 1 (not at all complete) to 5 (completely complete).

Mean accuracy scores (Likert scale, 1–5) for patient education materials generated at a seventh-grade reading level by ChatGPT, Google Gemini, and Microsoft Copilot were 4.60 ± 0.8, 4.55 ± 0.8, and 4.20 ± 0.9 (Table 5). Mean completeness scores for these materials were 4.10 ± 0.8, 4.00 ± 0.7, and 3.80 ± 0.9, respectively (*F* = 0.77, *P* = .466) (Table 5). There were no significant differences in accuracy or completeness among the 3 large language models when comparing patient education materials generated at an unspecified reading level with those generated at the seventh-grade reading level. Table 5 displays the accuracy and completeness scores for patient education materials generated by the large language models at various requested reading levels. When asked to identify inaccuracies and notable omissions in the patient education materials generated at the seventh-grade reading level, panelists most frequently noted the omission of information regarding long-term surveillance. In contrast, concerns regarding the use of medical jargon were substantially less common when a seventh-grade reading level was requested.

When expert panelists were shown patient education materials generated by all 3 large language models in a random side-by-side manner and asked to select the version they would most likely distribute to patients, they selected ChatGPT-generated materials 82% of the time, followed by Google Gemini (13%) and Microsoft Copilot (5%).

## Conclusions

This study evaluated the ability of large language models to answer patient questions and generate patient education materials related to uncommon retinal conditions. Our results demonstrate that, overall, all 3 large language models produced responses to patient questions that were both accurate and complete. However, the default reading level of these responses was substantially higher than what is recommended by the American Medical Association for the seventh-grade reading level, potentially limiting patient comprehension. The large language models examined in this study also demonstrated the ability to generate accurate and complete patient education materials for uncommon retinal conditions. Furthermore, when prompted to target a lower reading level, the models were able to successfully reduce the reading level of the educational materials without compromising accuracy. These findings suggest that large language models may be useful for tailoring patient education materials to patients with varying levels of health literacy.

Large language models that provide accurate information about uncommon retinal conditions represent an underutilized resource for retina specialists seeking to enhance patient understanding of their condition. The ability of large language models to generate high-quality educational materials that can be comprehended by patients with varying health literacy levels may help address disparities in medical literacy and support informed decision-making in eye care.

Our findings contribute to a growing body of literature examining the application of large language models in ophthalmology.^28^ Several prior studies have evaluated the ability of large language models to accurately respond to ophthalmology-related patient questions.^8–11,26,29,30^ However, findings regarding the accuracy of responses generated by large language models have been mixed (Table 6).^8,9,10,26,29,31,32^

**Table 6.** Comparison of Readability and Accuracy Findings from Previous Studies Evaluating Large Language Models in Ophthalmology.

Reference	Topic	Large Language Models/Search Engines Evaluated	Accuracy/Safety Findings	Readability Findings
Bernstein et al, 2023^9^	Mixed ophthalmology frequently asked questions	ChatGPT-3.5	No difference in accuracy between large language model-written and human-written responses; no difference in likelihood of possible harm; several incorrect or incomplete responses	Not assessed
Cappellani et al, 2024^10^	General ophthalmic diseases and management	ChatGPT-3.5	Multiple inaccuracies; potential risk for patient misinformation	Not assessed
Cohen et al, 2024^11^	Oculoplastics frequently asked questions + LLM-generated patient education materials	ChatGPT-3.5, Google Web Search	ChatGPT responses to patients’ frequently asked questions were more accurate and safer than Google Search responses	Default readability for ChatGPT responses to patients’ frequently asked questions and large language model-generated patient education materials was approximately at the 16^th^-grade level
Cohen et al, 2024^26^	Glaucoma frequently asked questions and LLM-generated patient education materials	ChatGPT-3.5, Google Search	ChatGPT responses to patients’ frequently asked questions more accurate than Google Search responses	ChatGPT responses to patients’ frequently asked questions were at approximately the 14^th^-grade level; patient education materials were at approximately 17^th^-grade level by default
Cohen et al, 2024^8^	Cataract and cataract surgery frequently asked questions and LLM-generated patient education materials	ChatGPT-3.5, Google Search	ChatGPT responses were significantly more accurate, especially for surgical counseling	ChatGPT responses to patients’ frequently asked questions were at approximately the 15^th^-grade level; Google Search responses were at approximately the ninth-grade level; postoperative instructions generated by ChatGPT were at the seventh-grade level
Tailor et al, 2025^30^	Neuro-ophthalmology questions	ChatGPT-3.5, ChatGPT-4, Claude 2, Bing, Bard	ChatGPT-4 and ChatGPT-3.5 achieved the highest quality and empathy scores; Expert-edited large language model responses achieved the highest quality and empathy ratings	Not assessed
Momenaei et al, 2023^35^	Surgical treatment of retinal diseases	ChatGPT-4	80%–90% of responses were consistently appropriate	Mean Flesch–Kincaid grade levels ranged from 10 to 14 for educational materials related to retinal detachment, macular holes, and epiretinal membranes
Strzalkowski et al, 2024^40^	Retinal detachment	ChatGPT-4 and Google Gemini	ChatGPT-4 provided more correct responses to complex questions with fewer serious errors; ChatGPT-4 outperformed Google Gemini in 8 of 13 questions	Google Gemini responses were easier to understand, although both models generated materials requiring a college-level reading ability

The findings of the present study support the potential of contemporary large language models to provide accurate and complete responses to patient questions. Our findings differ from those of some previous studies reporting inaccurate or incomplete large language model information, which may be attributable, in part, to the use of the most recent versions of 3 widely used large language models, whereas prior studies evaluated earlier model versions.^10,33–35^ It is also possible that the availability and quality of online educational resources influence the performance of large language models. Although the retinal conditions examined in this study are individually uncommon, with most conditions affecting only a small number of patients worldwide, variability in the quantity and quality of available online educational resources may have influenced the accuracy and completeness of large language model-generated responses. Further research is warranted to better understand the factors underlying the discrepancies among published studies.

Large language models that demonstrate the ability to provide accurate responses to patients’ frequently asked questions may have important implications for how ophthalmologists educate patients and how patients learn about their eye conditions. Currently, patients seeking health information online often turn to Google and other search engines, where they must navigate multiple sources and determine which information is accurate.^36,37^ Large language models may help address this challenge by providing direct, conversational responses to patient queries. As a result, large language models that provide reliable information have the potential to drastically streamline the health information-seeking process by providing timely, accessible responses tailored to individual patient queries. This is particularly important for uncommon retinal conditions, for which high-quality patient educational resources are often limited.^17–19^ It is especially important that information provided by large language models regarding uncommon retinal conditions is accurate, because these conditions are frequently managed by highly specialized retina specialists, with whom it may be difficult to obtain an appointment.

Although large language models cannot replace actual evaluation by an ophthalmologist, they may serve as a valuable adjunctive resource for patients awaiting an appointment with a specialist or those seeking information between clinic visits.

Although our study findings suggest that, on average, responses to patients’ frequently asked questions generated by large language models regarding uncommon retinal conditions are accurate, expert panelists noted some inaccuracies. These findings underscore the importance of exercising caution when using large language models as sources of medical information and highlight the need for continued oversight by eye care professionals. Notably, previous studies evaluating more common ophthalmic conditions have reported that large language model-generated responses to frequently asked questions may outperform information obtained through standard Google searches with respect to accuracy and usefulness.^8,11^ Therefore, when patients seek information regarding uncommon retinal conditions, large language models may serve as a useful starting point for obtaining background information and improving their understanding of their disease.

However, large language model-generated content should not be viewed as a substitute for information provided by ophthalmologists or authoritative patient education resources. Although the present study suggests that ChatGPT and Google Gemini can provide accurate foundational information regarding uncommon retinal conditions, directing patients to subspecialty-approved resources from trusted organizations such as the American Academy of Ophthalmology or the American Society of Retina Specialists remains an important component of patient education.

The ability to generate accurate and complete patient education materials at specified reading levels upon request has the potential to dramatically improve patient education for uncommon retinal conditions. Our results demonstrate that, by default, patient education materials generated by large language models are often written at a reading level that may be difficult to understand for many patients. However, this study also showcases the ability of large language models to improve the reading level of patient education materials upon request without compromising accuracy.

All 3 large language models demonstrated the ability to successfully reduce the reading level of patient education materials from a college level to a high school level. Such improvements in readability may significantly increase the proportion of patients capable of comprehending the information provided, as approximately 40% of Americans hold a college degree, whereas nearly 90% have completed high school.^38,39^ Notably, Google Gemini achieved the greatest reduction in grade level among the 3 models, indicating superior performance in optimizing readability while maintaining accuracy. This finding aligns with a prior study examining large language model-generated patient education materials associated with retinal detachment.^40^

Making patient education materials more comprehensible is clinically important because prior research in ophthalmology indicates that providing patients with understandable information about their condition can enhance disease understanding, facilitate informed decision-making, and ultimately improve patient outcomes.^41–43^

The ability of large language models to both generate organic patient education materials and revise existing content highlights one of the many potential applications of these tools in clinical practice. Two commonly cited barriers to the adoption of large language models among healthcare providers are concerns regarding the generation of false information (“hallucinations”) and unfamiliarity with the technology required to use these systems.^44–46^ With regard to concerns regarding hallucinations, our study found that responses to patients’ frequently asked questions and organically created patient education materials for uncommon retinal conditions were, on average, rated between “accurate” and “very accurate.” In addition, concerns regarding the technical complexity of large language models may be less substantial than perceived. In this study, generating accurate and comprehensible patient education materials required only basic user interaction with commercially available large language model platforms.

Taken together, these findings suggest that the practical barriers to incorporating large language models into patient education workflows may be lower than commonly assumed. As demonstrated in this study, large language models may offer an efficient means of developing patient education materials to improve readability while maintaining accuracy, potentially enhancing patient understanding with relatively limited time investment from clinicians.

This study has several limitations. First, we evaluated 10 relatively uncommon retinal conditions and analyzed the 5 most common frequently asked questions associated with each condition. As such, this sample represents only a subset of uncommon retinal diseases and potential patient questions. However, our goal was to assess large language model performance in generating patient-facing information for a group of relatively less common retinal conditions that are underrepresented in existing research. Consequently, the performance of large language models for other less common retinal conditions not included in this study may differ.

Second, frequently asked questions were solely obtained through Google searches. Although searches performed using other search engines, such as Bing, yielded many similar questions, the specific frequently asked questions identified may vary depending on the search engine used. Nevertheless, the primary aim of this study was to provide a broad overview of the ability of large language models to educate patients about uncommon retinal conditions.

Third, the evaluation of accuracy and completeness was based on ratings provided by retina specialists and is therefore inherently subjective. As a result, these assessments may not reflect the opinions of all retina specialists. However, interrater agreement among panelists was strong (intraclass correlation coefficient >0.75), suggesting a high degree of consistency in the evaluations. Although it cannot be assumed that the participating experts are fully representative of all retina specialists, the observed level of agreement supports the reliability of the scoring process.

Furthermore, readability indices were used as a proxy for the understandability of patient education materials. Although readability and comprehensibility are related, they are not synonymous, and it is possible that text rated as “highly readable” may still be difficult to understand by some patients. Nonetheless, the validated readability indices used in this study have been widely applied in ophthalmology and other medical specialties as practical measures of the understandability of patient education materials.^35,47–51^

In addition, although the large language models were instructed to generate patient education materials at a seventh-grade reading level according to the American Medical Association recommendations, it is possible that an even lower reading level is necessary to optimize understandability among the general public. Furthermore, our analysis was limited to a predefined subset of widely accessible, commercially deployed large language models that were available and integrated into common productivity workflows during the study period. Therefore, other contemporary models (eg, Claude, Grok, and DeepSeek) were not evaluated. As a result, our findings may not be generalizable to all currently available large language models, particularly those with different architectures, training data, or deployment contexts. Finally, although vitreoretinal specialists evaluated responses for overall quality and accuracy, our analysis did not specifically quantify the presence of potentially unsafe or misleading information related to therapeutic interventions, such as descriptions of treatments that may carry meaningful risks, limited supporting evidence, or uncertain clinical benefit. Similarly, although expert reviewers assessed responses for overall quality, we did not systematically categorize specific types of informational deficiencies, including unnecessary detail, omission of key clinical considerations, or distortion of established evidence. Future studies may benefit from more granular analyses of these potential issues.

Future studies should directly compare large language model-generated educational materials with existing patient education resources to evaluate relative readability, accuracy, and completeness. Such head-to-head benchmarking represents an important next step beyond the scope of the present study. Additional investigations could examine the impact of source material on the quality and readability of large language model-generated materials. For example, comparing educational materials generated from different sources, such as textbooks and other existing patient education materials, may help elucidate why the default reading level of large language model-generated materials is far above the recommended reading level.

Finally, incorporating patient perspectives into the evaluation of large language model-generated education materials can help to ensure that large language model resources align with real patient needs, improve comprehension, and address the questions and concerns most relevant to individuals living with uncommon retinal conditions.

In conclusion, our study demonstrates the ability of 3 large language models to generate accurate and complete responses to patients’ frequently asked questions regarding uncommon retinal conditions, expanding on prior research on the use of large language models for patient education in more common retinal conditions. Furthermore, our findings highlight the potential of large language models to both generate organic patient education materials and improve the readability of existing patient education materials for patients with varying health literacy levels. By facilitating access to accurate and comprehensible information, large language models may help address information gaps that can prevent patients from making informed decisions in eye care. A better understanding of the capabilities, limitations, and potential applications of large language models in ophthalmology may help guide their responsible integration into clinical practice.
